# A dysbiotic mycobiome dominated by *Candida albicans* is identified within oral squamous-cell carcinomas

**DOI:** 10.1080/20002297.2017.1385369

**Published:** 2017-10-27

**Authors:** Manosha Perera, Nezar Noor Al-hebshi, Irosha Perera, Deepak Ipe, Glen C. Ulett, David J. Speicher, Tsute Chen, Newell W. Johnson

**Affiliations:** ^a^ School of Dentistry and Oral Health, Griffith University, Queensland, Australia; ^b^ Maurice H. Kornberg School of Dentistry, Temple University, Philadelphia, USA; ^c^ Preventive Oral Health Unit, The National Dental Hospital (Teaching), Colombo, Sri Lanka; ^d^ School of Medical Science, Griffith University, Queensland, Australia; ^e^ Menzies Health Institute Queensland;Griffith University, Nathan, Australia; ^f^ Microbiology and Infectious Diseases, St. Joseph’s Healthcare Hamilton, Hamilton, Canada; ^g^ Department of Pathology and Molecular Medicine, McMaster University, Hamilton, Canada; ^h^ Department of Microbiology, Forsyth Institute, Cambridge, USA; ^i^ Dental Institute, King’s College London, London, United Kingdom

**Keywords:** Fungi, mycobiome, carcinoma, high-throughput nucleotide sequencing, DNA ribosomal spacer, microbiome, mouth, squamous cell

## Abstract

The aim of this study was to characterize the mycobiome associated with oral squamous-cell carcinoma (OSCC). DNA was extracted from 52 tissue biopsies (cases: 25 OSCC; controls: 27 intra-oral fibro-epithelial polyps [FEP]) and sequenced for the fungal internal transcribed spacer 2 region using Illumina™ 2 x300bp chemistry. Merged reads were classified to species level using a BLASTN-algorithm with UNITE’s named species sequences as reference. Downstream analyses were performed using QIIME™ and linear discriminant analysis effect size. A total of 364 species representing 160 genera and two phyla (Ascomycota and Basidiomycota) were identified, with *Candida* and *Malassezia* making up 48% and 11% of the average mycobiome, respectively. However, only five species and four genera were detected in ≥50% of the samples. The species richness and diversity were significantly lower in OSCC. Genera *Candida*, *Hannaella*, and *Gibberella* were overrepresented in OSCC; *Alternaria* and **Trametes** were more abundant in FEP. Species-wise, *Candida albicans*, *Candida etchellsii*, and a *Hannaella luteola*–like species were enriched in OSCC, while *a* *Hanseniaspora uvarum*–like species, *Malassezia restricta*, and *Aspergillus tamarii* were the most significantly abundant in FEP. In conclusion, a dysbiotic mycobiome dominated by *C. albicans* was found in association with OSCC, a finding worth further investigation.

## Introduction

Oral squamous-cell carcinoma (OSSC) accounts for >90% of cases of oral cancer. OSSC is a malignancy with poor prognosis, representing a public health challenge, particularly in less developed regions where it ranks as the 8th most common cancer type [1–3]. The incidence rates are exceptionally high in certain countries such as Papua New Guinea, India, Sri Lanka, the Maldives, and Pakistan [1]. There are a number of well-established risk factors for OSSC, including smoking, smokeless tobacco usage, areca nut and betel quid chewing, alcohol consumption, and human papillomavirus infections [4,5]. However, around 15% of oral cancer cases are not attributable to any of these. The role of other factors such as infection and inflammation is thus emerging [6,7].

The oral cavity harbors the second most diverse microbiota in our body: the oral microbiome [8]. Oral microorganisms, predominantly bacteria, form homeostatic communities that live in a mutualistic relationship with the host. However, local ecological challenges may disturb the community balance and result in a microbial ‘dysbiosis’ characterized by altered microbiome profile and the potential to cause or contribute to disease [9]. Technological advances, particularly the advent of next-generation sequencing (NGS), have revolutionized the study of the oral microbiome in health and disease [10]. Periodontitis and dental caries, the most common oral diseases, are recognized today as sequelae of oral microbial dysbiosis [9,11]. Recent evidence from NGS studies also suggest a possible role of a dysbiotic microbial community in OSCC [7,12]. However, all these studies have focused only on the bacterial component of the microbiome (i.e. the bacteriome).

Numerous fungi are found in the oral cavity, with the genus *Candida* being the most commonly isolated and thus studied. Recent studies using NGS have revealed the presence of a complex fungal community (the mycobiome) in healthy individuals. In 2010, a study by Ghannoum et al. revealed the presence of a core oral mycobiome consisting of 13 taxa, with *Candida*, Saccharomycetales, *Cladosporium*, and *Aspergillus* being the most abundant [13]. In 2014, Dupuy et al. [14] identified an additional five core genera, of which *Malassezia* was detected for the first time in the oral cavity and was even found to be more abundant than *Candida* in some of the subjects. So far, no attempts have been made to explore the potential role of the oral mycobiome in oral health and disease, including oral cancer. This is surprising, given the existing evidence implicating *Candida albicans* in oral carcinogenesis [15].

The objective of this study was to use NGS coupled with a species-level taxonomy assignment algorithm to compare the mycobiome profile within OSSC tissues to benign intra-oral fibro-epithelial polyps (FEP) and to identify fungal taxa that may play a role in oral carcinogenesis.

## Methods

### Study design, setting, and subjects

This was a case-control study. Subjects were recruited between 17 April 2015 and 2 August 2015 at nine oral and maxillofacial (OMF) units in six provinces of Sri Lanka. Cases comprised 25 Sinhala, ≥40-year-old males with histologically confirmed OSCC affecting the buccal mucosa or tongue. The control group consisted of 27 Sinhala males with a clinical diagnosis of FEP also involving the buccal mucosa or tongue. Subjects with a history of antibiotic use in the last 2 months were excluded.

Ethical approval for the study was obtained from the Faculty Research Committee, Faculty of Dental Sciences, University of Peradeniya, Sri Lanka (FRC/FDS/UOP/E/2014/32) and Griffith University Human Research Ethics Committee, Australia (DOH/18/14/HREC). Written informed consent was obtained from each participant.

### History taking and clinical examination

Data were collected using a pretested, interviewer-administered questionnaire, which comprised information on socio-demographics and risk habits, including use of smokeless tobacco, areca nut and betel quid chewing, tobacco smoking, and alcohol consumption. Clinical oral examinations were conducted by dental public-health specialists. The oral mucosa was thoroughly inspected for any growth, ulceration, or white patches. The number of missing teeth was recorded. Oral hygiene status was assessed with the simplified oral hygiene index [16], while periodontal status was assessed using bleeding on probing (BOP), periodontal pocket depth (PPD), and clinical attachment loss (CAL) at four sites per anterior tooth and six sites per posterior tooth.

### Tissue sampling and DNA extraction

For suspected OSSC cases, tissue samples were obtained from the incisional biopsies taken for diagnosis. The freshly taken biopsy was laid on a pile of sterile gauze, and a small piece of tissue (~3 mm^3^) was excised from the deep tissue at the macroscopically visible advancing front of the neoplasm, avoiding contamination from the tumor surface. A new sterile surgical blade was used for each case. The sample was aseptically transferred into a screw-cap vial and placed in a polystyrene box containing dry ice. These were transferred to a −80°C freezer in a university laboratory as soon as practicable. Simultaneously, the rest of the biopsy was sent in 10% buffered formalin for histopathological diagnosis. Only samples histopathologically confirmed as OSSC were included in the study. Control tissue samples were obtained as above from freshly excised, clinically diagnosed FEPs.

Tissue samples (~100 mg each) were finely chopped using a sterile blade. DNA extraction was then performed using Gentra Puregene Tissue kit (Qiagen, Hilden, Germany), according to the manufacturer’s instructions (solid tissue protocol) with a few modifications: (1) incubation in the lysis buffer was performed overnight; and (2) an additional lysis step using 50 units of mutanolysin at 37°C for 1.5 h to digest the cell wall of Gram + bacteria was included prior to the addition of Proteinase K (the samples had also been planned to be analyzed for bacterial content). Total DNA concentration and purity were determined using the NanoDrop™ 1000 Spectrophotometer (Thermo Fisher Scientific, Waltham, MA). The extracts were stored at –80°C.

### Fungal load

The fungal load was assessed by quantification of the ribosomal internal transcribed spacer 2 (ITS2) normalized to the human β-actin gene using SYBR Green-based real-time polymerase chain reaction (PCR) and the 2^–ΔΔ^ ^Ct^ method. The primers ITS3-F (5ʹ-GCATCGATGAAGAACGCAGC-3ʹ) and ITS4-R (5ʹ-TCCTCCGCTTATTRATATGC-3ʹ) [17] were used to target ITS2, while primers β-actin-gDNA-F (5'-TCCGCAAAGACCTGTACGC-3ʹ) and β-actin-gDNA-R (5ʹ-CAGTGAGGACCCTGGATGTG-3ʹ) were used to amplify the β-actin gene. Each reaction comprised 5 µL SYBR Green mastermix, 0.2 µL primer mix (10 µM), 2.8 µL water, and 2 µL template DNA diluted 1:320. Amplification was carried on a ViiA7 platform (Applied Biosystems, Foster City, CA), including an initial enzyme activation cycle at 95°C for of 10 min followed by 50 cycles of denaturation at 95°C for 15 s, annealing at 55°C for 30 s, and extension at 60°C for 60 s.

### Amplicon library preparation and sequencing

An amplicon library was prepared as per the Illumina’s protocol, except that amplification was performed with the Q5 Hot Start High-Fidelity 2× MasterMix (New England Biolabs, Ipswich, MA). In brief, ITS2 was amplified using the primers ITS3-F and ITS4-R, linked to Illumina’s specific adapter sequences in standard PCR conditions. The resultant PCR amplicons (~250–590 bp) were purified using Agencourt AMPure XP beads (Beckman Coulter, Brea, CA). A second PCR was performed to tag the amplicons with unique eight-base barcodes using Nextera XT v2 Index Kit sets A-D (Illumina, San Diego, CA). A set of negative amplification controls (mastermix alone and with other reaction components) were included for both the amplicon production and indexing reactions. The tagged amplicons were then pooled together in equimolar concentrations and sequenced on a MiSeq Sequencing System (Illumina) using v3 2 × 300bp, paired-end sequencing chemistry in the Australian Centre for Ecogenomics, according to the manufacturer’s protocol.

### Processing of sequencing data

Raw sequencing data were deposited in (and are publicly available from) Sequence Reads Archive (SRA) under project no. PRJNA375780. Reads with primer mismatches were removed, and primer sequences were trimmed off. Paired sequences were then merged with PEAR [18] using the following parameters: minimum amplicon length, 213 bp; maximum amplicon length, 552 bp; and *p*-value, 0.001. Preprocessing of the merged reads was performed using mothur v1.38.1 [19]. First, to minimize sequencing errors stringently, reads with ambiguous bases, reads with homopolymers >8 bp, or reads that did not achieve a sliding 50-nucleotide Q-score average of ≥30 were filtered out. Second, the high-quality reads were cleared of chimeras with Uchime [20] using the self-reference approach [21]. Finally, sequences representing non-fungal lineages, identified by preliminary taxonomy using mothur’s classify.seqs command, were removed.

### Taxonomy assignment algorithm and downstream analysis

The high-quality, non-chimeric merged reads were classified at the species level employing a previously described BLASTN-based algorithm, modified to analyze the fungal ITS2 region instead of the bacterial 16S rRNA gene [22]. A set of 23,423 fungal ITS sequences representing all named species (16,595 species) in UNITE’s database v7.1 (https://unite.ut.ee/repository.php; 22 August 2016 dynamic release; untrimmed sequences) [23] was used as reference (the fasta and taxonomy files of this set can be downloaded at ftp://www.homd.org/publication_data/20170221/). Briefly, the reads were individually BLASTN searched against the reference set at an alignment coverage of ≥99% and a percent identity of ≥98.5%. Hits were ranked by percent identity and, when equal, by bit score. Reads were assigned taxonomies of the best hits. Reads with the best hits representing more than one species were screened again for chimeras using a *de novo* check at 98% similarity with USEARCH v8.1.1861 and, if not chimeric, were assigned multiple-species taxonomy [24]. Reads with no matches at the specified criteria underwent secondary *de novo* chimera checking as above, and then *de novo*, species-level operational taxonomy unit (OTU) calling at 98% using USEARCH. Singleton OTUs were excluded; the rest were considered potentially novel species, and a representative read from each was BLASTN-searched against the same reference sequence set again to determine the closest species for taxonomy assignment.

Downstream analysis was performed, as previously described [22]. In short, Quantitative Insights Into Microbial Ecology (QIIME™) v1.9.1 [25] was employed to perform further analysis, including generation of taxonomy plots, rarefaction, calculation of species richness and diversity indexes, computing distance matrixes, and running principle component analysis (PCoA). Detection of differentially abundant taxa between the cases and controls was done using linear discriminant analysis effect size (LEfSe) [26].

## Results

Four samples ended up with a low read count (<3,000) and one with very high count (an outlier) and were thus excluded. Results are presented hereafter for 22 cases and 25 controls. The clinical characteristics of both groups are presented in Table 1. The cases, albeit older on average than controls, were heavier users of betel quid and alcohol, and smoked more, these being the traditional risk factors for oral cancer. They had significantly poorer oral hygiene, fewer teeth, and more severe periodontal disease.

**Table 1.** Clinical characteristics of the study subjects.

Variable	Cases (*n* = 22)	Controls (*n* = 25)
Age (*M* ± *SD*)*	61.00 ± 9.5	50.58 ± 13.5
% males	100	100
Betel quid: *n* (%)**		
Never	0 (0.0)	4 (16)
Past	3 (13.6)	2 (08)
Sometimes	1 (4.6)	7 (28)
Daily	18 (81.9)	12 (48)
Smoking: *n* (%)**		
Never	4 (18.2)	8 (32)
Past	5 (22.7)	7 (28)
Sometimes	4 (18.2)	4 (16)
Daily	9 (40.9)	6 (24)
Alcohol use: *n* (%)**		
Never	0 (0.0)	4 (16)
Past	5 (22.7)	2 (8)
Sometimes	4 (18.2)	15 (60)
Daily	13 (59.1)	4 (16)
Oral hygiene: *n* (%)**		
Bad	6 (27.3)	3 (12)
Fair	14 (63.6)	10 (40)
Good	2 (9.1)	12 (48)
Missing teeth (*M* ± *SD*)*	14.73 ± 10.01	3.85 ± 4.82
Periodontal status: *n* (%)**,^a^		
Severe periodontitis	8 (36.4)	2 (08)
Moderate periodontitis	11 (50.0)	6 (24)
No or mild periodontitis	3 (13.6)	17 (68)
Site affected: *n* (%)		
Tongue	14 (63.6)	19 (76)
Buccal mucosa	8 (36.4)	6 (24)
Histopathology: *n* (%)		
Well-differentiated SCC	11 (50)	–
Moderately differentiated SCC	11 (50)	–
Fibro-epithelial polyp	–	25 (100)

^a^Classification made according to Page and Eke [27].

*p ≤ 0.05 (*t*-test); **p ≤ 0.05 (chi-square test).

SSC, squamous-cell carcinoma.

### DNA extracts, fungal load, and sequencing/data processing statistics

Total DNA extracted averaged 482.08 ng/µL (range 16.85–1,908 ng/µL; <100 ng/µL from 8/47 [17%] samples), with all samples containing DNA of high purity (average 260/280 ratio was 1.93; range 1.81–2.07). However, the mean 260/230 ratio was 1.54 (range 0.41–2.22; 260) indicating the presence of inhibitors in some of the samples, an issue that was dealt with using dilution before amplification. The average fungal load was 2.4 ITS copies per 1,000 β-actin gene copies (range 0.06–135). This did not significantly differ between the cases and controls.

A few sequences were detected in the negative control, indicating very low background noise (see Supplementary DataSet 1). Sequencing generated 1,576,427 raw paired reads: 13.6% of these were discarded due to primer mismatches, and 97.4% of the remaining reads were successfully stitched with PEAR. Quality filtration and chimera checking removed 7.9% of the merged reads, thus leaving 1,063,430 reads (67.5%), 205–535 bp long. Of these, 1,017,131 reads (95.7%) were successfully classified to the species level; 2.3% did not return BLASTN matches, and 2% formed singleton OTUs and were excluded. The number of classified reads per sample averaged 21,641 (range 3,973–54,849).

### Overall mycobiome profile

A total of 364 species belonging to 162 genera and two phyla were detected in the samples. The relative abundances and detection frequencies of these taxa in each of the samples and across the study groups are presented in Supplementary Data Sets 2–4. The number of species per sample ranged from 4 to 64. However, only 74 genera and 125 species were identified in more than one sample: seven genera and 10 species in ≥25% of the samples, and four genera and five species in >50%.

**Figure 1.**
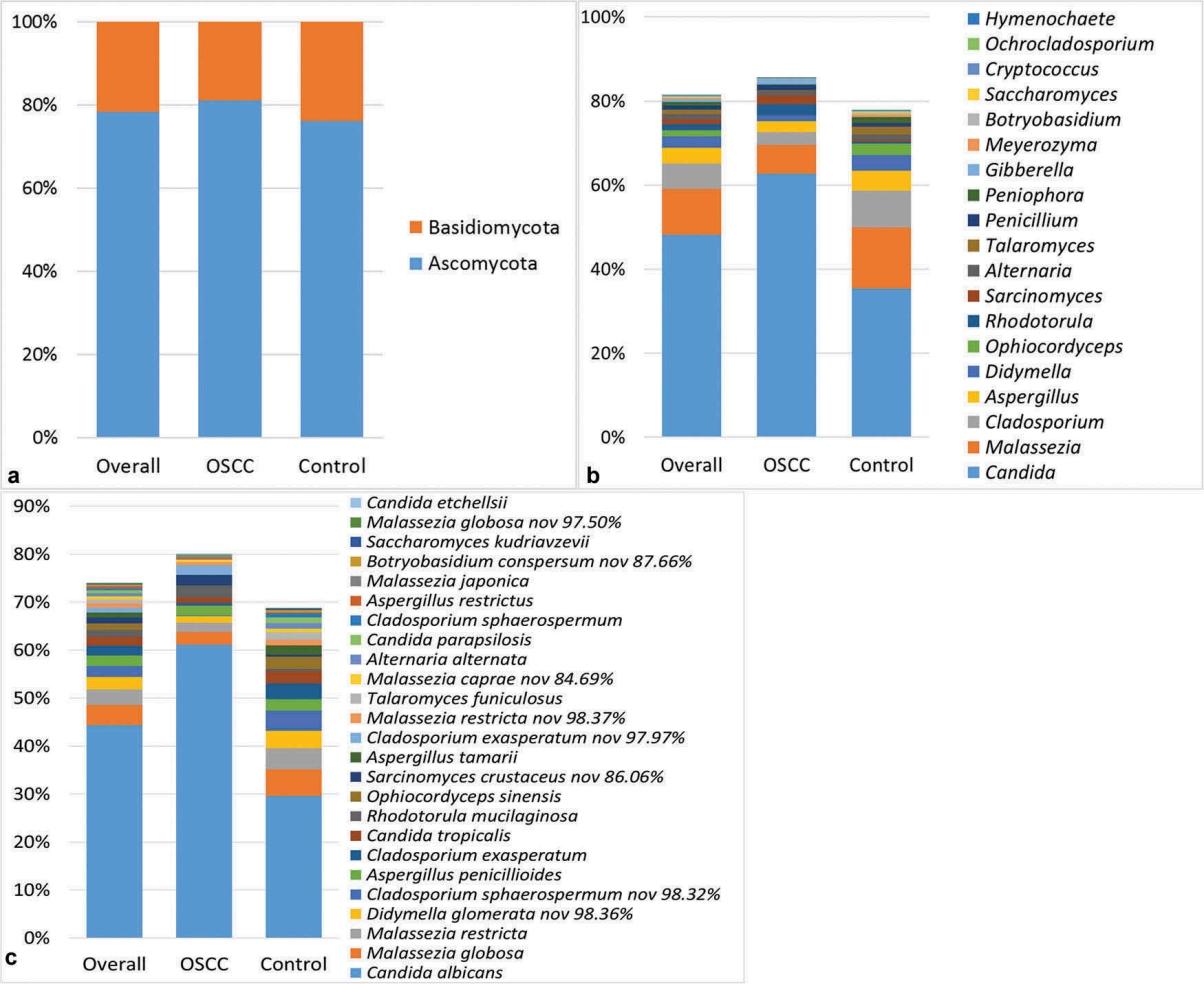
Mycobiome profile. Average relative abundances of the two phyla identified (a) as well as the genera (b) and species (c) detected in ≥15% of the samples.

The relative abundances of the two phyla identified, as well as the genera and species detected in ≥15% of the samples, are shown in Figure 1. On average, the phyla Ascomycota and Basidiomycota accounted for 78.4% and 21.6% of the mycobiome, respectively. At the genus level, *Candida* was detected in 100% of the samples and constituted 48% of the average mycobiome. The genera *Malassezia, Cladosporium*, and *Aspergillus* were identified in ≥75% of the samples, with an average relative abundance of 11%, 6.1%, and 3.7%, respectively. With regard to species, *C. albicans* was found in all samples at a mean relative abundance of 44.4%. *Malassezia restricta, Aspergillus penicillioides*, and *Malassezia globosa* were identified in 83%, 70.2%, and 68.1% of the samples, respectively, and accounted for 3.2%, 2.2%, and 4.2% of the average mycobiome, respectively. *Cladosporium exasperatum* and a potentially novel species close to *Cladosporium sphaerospermum* were also identified in half of the samples at an average relative abundance of >2%.

However, the relative abundance of these taxa varied significantly between the samples. In addition, the mycobiome of some of the samples was dominated by taxa other than those mentioned above. Examples of species abundant in single samples include *Rhodotorula mucilaginosa, Sporidiobolus johnsonii, Penicillium toxicarium, Toxicocladosporium irritans, Gibberella intricans, Alternaria infectoria, Ophiocordyceps sinensis*, and *Aspergillus tamarii*, as well as a number of potentially novel taxa.

### OSCC versus FEP

**Figure 2.**
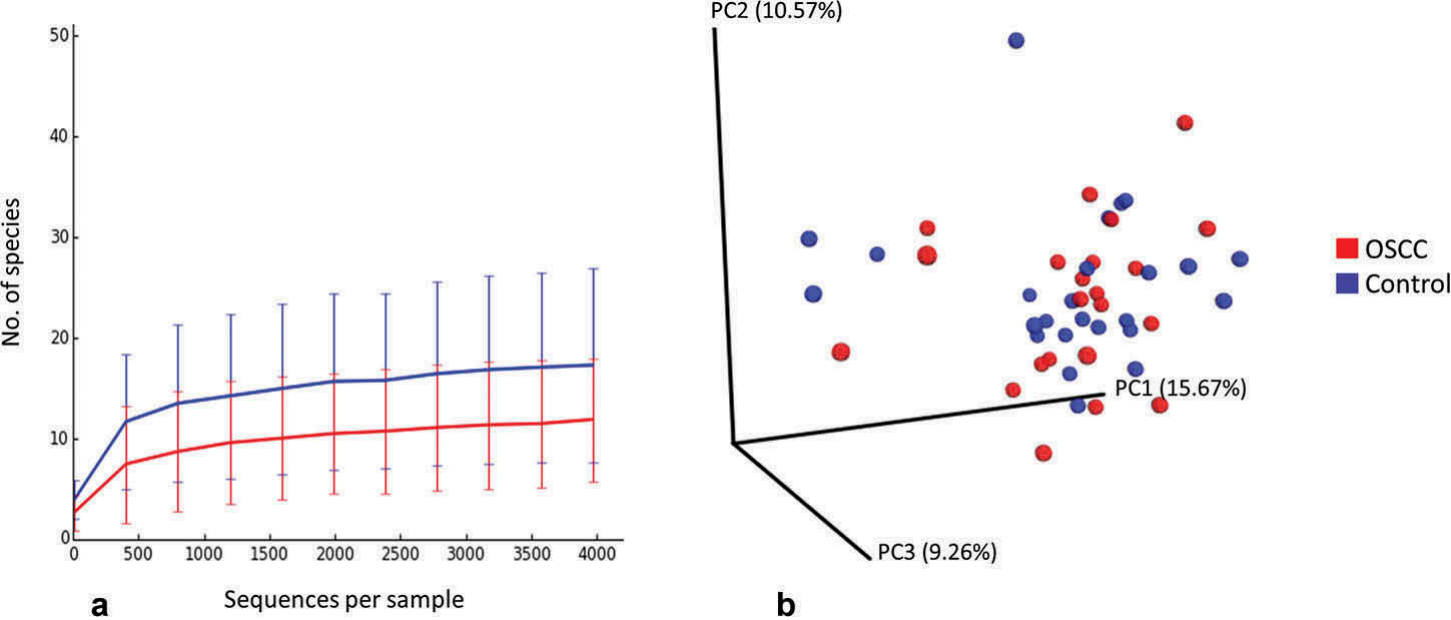
Rarefaction and β-diversity. (a) Rarefaction curves showing the number of observed species as a function of sequencing depth. (b) Non-clustering of the study subjects by principal components analysis (weighted Unifrac).

The number of species per sample ranged from 4 to 29 for the cases and from 8 to 64 for the controls. The FEP controls had significantly higher species richness and α-diversity than the cases (Table 2). Rarefaction curves show that as few as 1,500 reads per sample represented sufficient sequencing depth (Figure 2a). No separate clusters formed for the cases and controls by PCoA (Figure 2b).

**Table 2.** Species richness, α-diversity, and coverage (*M* ± *SE*) calculated from the rarefied biom.

Group	Observed richness*	Chao1*	Shannon index*	Good’s coverage
OSCC	11.7 ± 6.4	13.6 ± 7.0	1.5 ± 0.9	0.999 ± 0.000
FEP	17.7 ± 9.8	19.9 ± 12.2	2.1 ± 1.2	0.999 ± 0.001

*p ≤ 0.05 (Mann–Whitney test).

OSCC, oral squamous-cell carcinoma; FEP, fibro-epithelial polyps.

**Figure 3.**
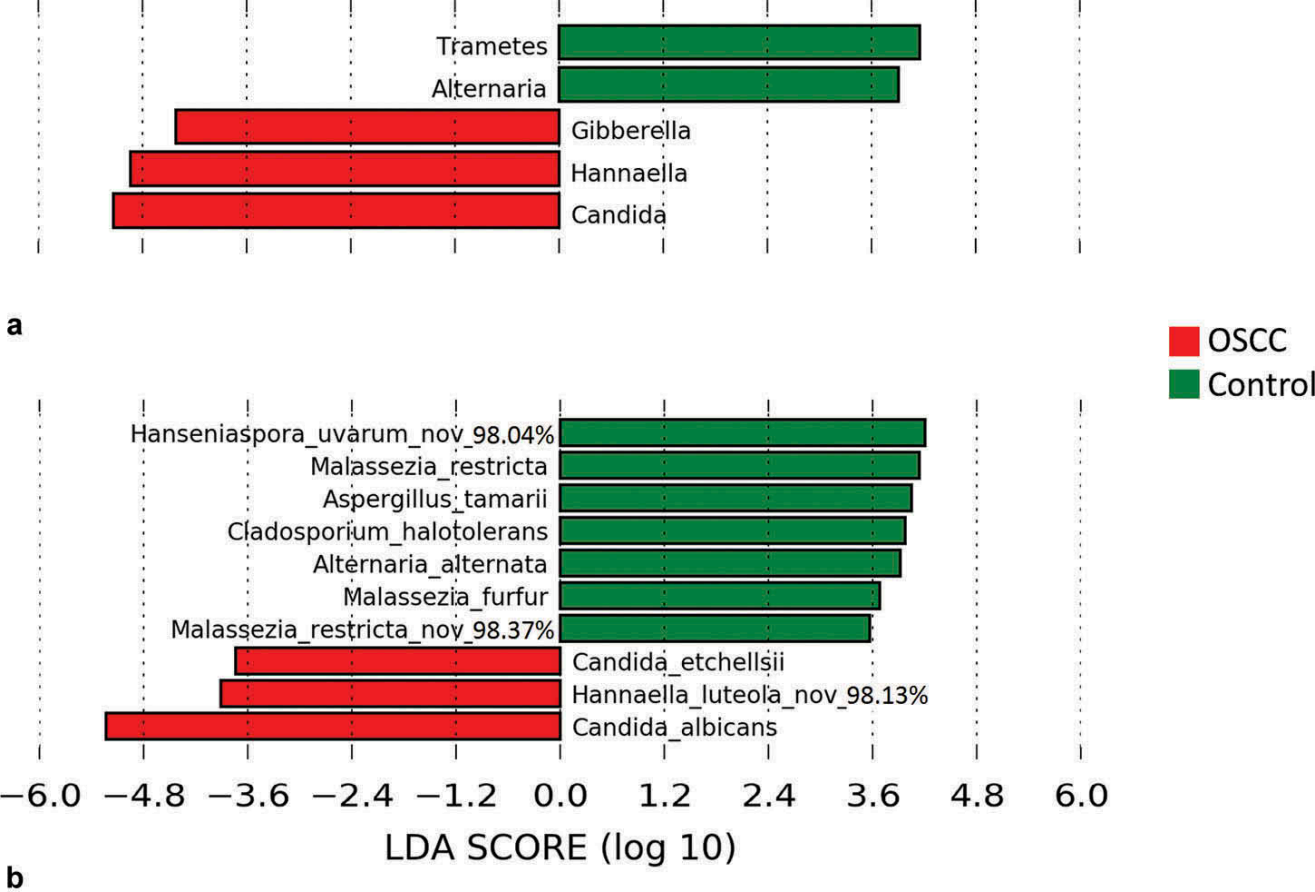
Differentially abundant taxa. Linear discriminant analysis effect size (LEfSe) analysis showing genera (a) and species (b) that were significantly differentially abundant between the cases and controls (LDA score ≥3). The differences were also found to be significant by the Mann–Whitney test.

The genera and species found by LEfSe to be differentially abundant between the cases and controls are shown in Figure 3. The genera *Candida, Hannaella*, and *Gibberella* were significantly more abundant in OSCC. In contrast, *Trametes* and *Alternaria* were strongly associated with FEP. At the species level, *C. albicans, C. etchellii*, and a potentially novel species close to *Hannaella luteola* were significantly enriched in OSCC. *C. albicans* was identified in 100% of the samples, but the average relative abundance in OSCC was twice that in the controls (61.2% vs. 29.6%). *C. etchellsii* was identified in 32% of the cases at a relative abundance of 0.006–2.56% compared to only 8% of the controls at a relative abundance of 0.007–0.12%. The *H. luteola*–like species was detected in 20% of OSCC samples versus none of the controls. On the other hand, a potentially novel *Hanseniaspora uvarum–*like species, in addition to *M. restricta, A. tamarii, Cladosporium halotolerans, Alternaria alternata*, and *Malassezia furfur* were overrepresented or even exclusively found in FEP (see Supplementary Table S1 for a list of taxa exclusively found in either group at a prevalence of ≥10%).

## Discussion

To the authors’ knowledge, this is the first study to explore the mycobiome associated with oral cancer. The study subjects were recruited from Sri Lanka, a developing lower-middle income country where oral cancer is the most common malignancy among men [1]. Deep-tissue biopsies, rather than surface swabs or saliva, were used to ensure that any association identified was more relevant to carcinogenesis. Unlike previous studies, OSCC was compared with FEP instead of buccal epithelium or adjacent normal tissue (i.e. malignant vs. benign growths). This is a unique approach that is arguably a strength of the study. However, since FEP is diagnosed at a younger age, a significant difference in age was inevitable. OSCC patients were also heavier users of tobacco and of betel nut and alcohol and had more severe periodontitis. These are all established risk factors of OSCC, and it would have been impossible to match the controls for them or to adjust for their confounding effects statistically, given the small sample size. Nevertheless, these factors may have accounted in part for the microbial differences observed—an inherent limitation that has to be acknowledged. There are other limitations to note. First, some DNA samples had low 260/230 ratios, suggestive of the presence of inhibitors. However, these samples amplified successfully after dilution. Second, the DNA extraction protocol was not optimized to maximize recovery of fungal DNA from the tissue samples, which may have resulted in underestimation of the fungal loads. Finally, a few reads were detected in the control samples, which, while having negligible effect on the results, suggested some sort of contamination at some stage of the laboratory work. The latter could have been due to contamination of the DNA extraction or sequencing reagents by extraneous organisms, now known to be a problem around the world [28].

Coupling Illumina’s 2 × 300 bp sequencing chemistry and merging of paired reads ensured that full ITS2 amplicons were obtained, maximizing taxonomic resolution. This, in addition to limiting the reference set to sequences from named species only, enabled the reads to be classified at the species level. A percent identity threshold of 98.5% (i.e. like that used to generate species hypothesis in UNITE’s dynamic release) and high query alignment coverage (≥99%) cutoff increased the reliability of taxonomic assignment and eliminated the possibility of forced classifications by BLASTN. However, this approach carries the risk of assigning reads belonging to species not represented in the reference set to another closely related species.

Of the species identified, 239/364 (65.6%) were from single samples, indicating that they represent transient environmental fungi. Although the scope of the current study is different from that of the previous two oral mycobiome studies in which salivary samples from healthy subjects were analyzed, some comparisons can be made. Ghannoum et al., using a relative abundance cutoff of 1%, identified 101 species in their samples [13]. Applying the same cutoff to our samples left 137 species, which is comparable. They also described a basal mycobiome comprising 13 taxa that were present in ≥20% of the subjects. Consistently, seven of these genera, namely *Candida, Aspergillus, Cladosporium, Alternaria, Cryptococcus, Gibberella,* and *Saccharomyces*, were detected at the same frequency in at least one of the two groups. Interestingly, *Malassezia*, which was not detected by Ghannoum et al., was identified in this study as the second most common and abundant genus after *Candida*. This substantiates the more recent findings by Dupuy et al., who, for the first time, described this genus in the oral cavity and even found it to be more abundant than *Candida* [14]. The same study described a core mycobiome comprising 14 core genera detected in ≥50% of subjects. Four of these (*Candida, Malassezia, Aspergillus*, and *Cladosporium*) were also found in more than half of our samples. Put together, these findings provide evidence for the existence of a resident, core oral fungal community.

Fungi in the oral cavity belong to what is called the rare biosphere [29]. It is therefore not surprising that they were detected in low counts, especially given that deep-tissue samples were analyzed. Regardless, their role in disease should not be discounted, as even in these small number, they can have a substantial effect on the microbiota, functioning as keystone pathogens [30]. There was, however, no significant difference in the fungal load between the cases and controls, which is inconsistent with the study by Berkovits et al. [31], who found the ‘fungal burden’ to be higher in OSCC patients compared to healthy controls. However, this may be explained, at least in part, by the fact that surface swabs rather than tissue samples were used in that study: more fungi are likely to be present on the rough tumor surface compared to the smooth surface of the healthy mucosa. In addition, the authors in that study used a culture-based approach rather than quantitative PCR, which may have resulted in missing some difficult-to-grow species. Indeed, *Malassezia*, which is a dominant taxon in many of the samples in this study, was not detected in the study by Berkovits et al. [31]. As discussed below, the differences between OSCC and FEP in this study were in terms of relative abundances.

*Candida*, specifically *C. albicans* and *C. etchellsii*, showed the strongest association with OSCC in the current study. In fact, candidiasis has for long been proposed as a risk factor for malignant transformation of oral potentially malignant disorders, including leukoplakia [32]. Some strains of *C. albicans* have high nitrosation potential and have been experimentally shown to induce dysplasia [33]. Recently, there has been increasing evidence of the association between candida infection and OSCC [15]. Nagy et al. recovered *C. albicans* from the surface of 8/21 (38%) neoplasms but from none of the control samples [34]. Similarly, Čanković et al. isolated *Candida* from 9/30 (four *C. albicans* and five non-*albicans*) cancer surfaces (30%) but from the surface of only 2/30 (6.6%) benign growths [35]. Gall et al. identified *Candida* spp. in 31/48 (65%) oral cancer cases, but the study did not include healthy control samples for comparison [36]. More recently, Berkovits et al. isolated yeasts, predominantly *Candida* spp., from the surface of 18/20 (90%) tumors compared to only 12/40 (30%) healthy mucosae [31]. In the present study, *C. albicans* was detected in 100% of the samples, which is probably a reflection of the much higher sensitivity of NGS compared to cultural techniques used in previous studies. The difference, however, between the cases and controls in the current material was in terms of relative abundance (average of 61.2% in the cases vs. 29.6% in the controls).

*C. etchellsii* was identified in 32% of the cases vs. 8% of the controls. This is the first time a specific *Candida* species other than *C. albicans* has been implicated in oral cancer, a finding worth further investigation. *Hannaella* and *Gibberella* spp. were also overrepresented in OSCC tissues. These are typically found on plants as commensals and pathogens, respectively [37,38]. Therefore, they probably represent contaminants of the OSCC rather than members of the oral mycobiome community, especially considering that they were found in only one control sample. However, their contribution to the carcinogenic process cannot be excluded. In fact, one *Gibberella* spp. (*G. moniliformis*) produces a mycotoxin that has been shown to induce liver cancer in rodents [39].

Species belonging to *Malassezia, Aspergillus, Alternaria, Cladosporium*, and *Hanseniaspora* were significantly enriched or exclusively found in the controls. *Malassezia* spp. are normal colonizers of healthy skin [40] and have been recently found to be dominant members of the salivary mycobiome [14]. *Aspergillus, Alternaria*, and *Cladosporium* have also been described as core oral fungal taxa [13,14]. Interestingly, some species of these genera, including *A. tamarii* and *A. alternata*, which were identified in the current samples, are known to produce compounds with anticancer activity [41–44]. In addition, statins produced by *A. tamarii* have been shown to inhibit growth of *C. albicans* [43], which possibly explains why all samples with high relative abundance of this species had very low levels of *C. albicans* (Supplementary Data Set 3). Therefore, while these species may simply represent transient environmental fungi or passenger oral fungal taxa, it is also possible that carriage of some of these species confers some protection against the development of oral cancer. Further research to explore these scenarios is needed to harness their potential for novel prevention and control strategies.

In conclusion, the current study uncovered a dysbiotic mycobiome characterized by lower species diversity and increased *C. albicans* relative abundance in association with OSCC. It also identified a number of fungal taxa capable of producing anticancer compounds exclusively in non-cancerous tissues. The relevance of these findings for development of risk markers and for prevention and control of OSCC warrants further investigation.

## Supplementary Material

Supplementary_data.zip
