# Supplementary material for: A dysbiotic mycobiome dominated by Candida albicans is identified within oral squamous-cell carcinomas
Source: J Oral Microbiol. 2017 Oct 27;9(1):1385369. doi: 10.1080/20002297.2017.1385369 (PMC5678454; doi:10.1080/20002297.2017.1385369)
Supplement: Supplementary_data.zip [file ZJOM_A_1385369_SM1553.zip › ST4. Taxa exclusively found in either group at 10%.docx]

**Table S4.** List of taxa exclusively identified in either group at prevalence ≥ 10%

| **Exclusively in OSCC** | **%** | **Exclusively in Controls** | **%** |
| --- | --- | --- | --- |
| *Hannaella luteola_nov_98.13%*  *Penicillium toxicarium*  *Malassezia slooffiae_nov_96.34%*  *Aureobasidium pullulans* | 18.2%  13.6%  13.6%  13.6% | *Aspergillus tamarii*  *Alternaria alternata*  *Malassezia furfur*  *Hanseniaspora uvarum_nov_98.04%*  *Talaromyces funiculosus*  *Candida salmanticensis_nov_79.52%*  *Talaromyces funiculosus_nov_98.36%*  *Neophysalospora eucalypti_nov_96.99%*  *Pseudorobillarda siamensis_nov_93.02%*  *Eremascus albus_nov_79.67%*  *multigenus multispecies_spp14_2*  *Cordana ellipsoidea_nov_93.96%*  *Talaromyces minioluteus_nov_95.58%*  *Gaeumannomyces radicicola_nov_91.28%*  *Didymella glomerata*  *Ophiostoma pulvinisporum_nov_89.67%*  *Aspergillus amstelodami*  *Alternaria multispecies_spp16_2*  *Aspergillus nomius* | 28.0%  28.0%  24.0%  20.0%  16.0%  16.0%  16.0%  16.0%  12.0%  12.0%  12.0%  12.0%  12.0%  12.0%  12.0%  12.0%  12.0%  12.0%  12.0% |
